# Microbial biocatalysis of quercetin-3-glucoside and isorhamnetin-3-glucoside in *Salicornia herbacea* and their contribution to improved anti-inflammatory activity

**DOI:** 10.1039/c9ra08059g

**Published:** 2020-02-03

**Authors:** Hyung Jin Ahn, Hyun Ju You, Myeong Soo Park, Zhipeng Li, Deokyeong Choe, Tony Vaughn Johnston, Seockmo Ku, Geun Eog Ji

**Affiliations:** Department of Food and Nutrition, Research Institute of Human Ecology, Seoul National University Seoul 08826 Republic of Korea geji@snu.ac.kr; Center for Human and Environmental Microbiome, Institute of Health and Environment Seoul 08826 Republic of Korea; Research Center, BIFIDO Co., Ltd. Hongcheon 25117 Republic of Korea; Fermentation Science Program, School of Agriculture, College of Basic and Applied Sciences, Middle Tennessee State University Murfreesboro TN 37132 USA seockmo.ku@mtsu.edu

## Abstract

*Salicornia herbacea* (glasswort) is a traditional Asian medicinal plant which exhibits multiple nutraceutical and pharmaceutical properties. Quercetin-3-glucoside and isorhamnetin-3-glucoside are the major flavonoid glycosides found in *S. herbacea*. Multiple researchers have shown that flavonoid glycosides can be structurally transformed into minor aglycone molecules, which play a significant role in exerting physiological responses *in vivo*. However, minor aglycone molecule levels in *S. herbacea* are very low. In this study, *Bifidobacterium animalis* subsp. *lactis* AD011, isolated from infant feces, catalyzed >85% of quercetin-3-glucoside and isorhamnetin-3-glucoside into quercetin and isorhamnetin, respectively, in 2 h, without breaking down flavonoid backbones. Functionality analysis demonstrated that the quercetin and isorhamnetin produced showed improved anti-inflammatory activity *vs.* the original source molecules against lipopolysaccharide induced RAW 264.7 macrophages. Our report highlights a novel protocol for rapid quercetin and isorhamnetin production from *S. herbacea* flavonoids and the applicability of quercetin and isorhamnetin as nutraceutical molecules with enhanced anti-inflammatory properties.

## Abstract 2

*Bifidobacterium animalis* subsp. *lactis* AD011 catalyzed >85% of quercetin-3-glucoside and isorhamnetin-3-glucoside into quercetin (Q) and isorhamnetin (IR), respectively, in 2 h. These Q and IR showed improved anti-inflammatory activity.

## Graphical abstract

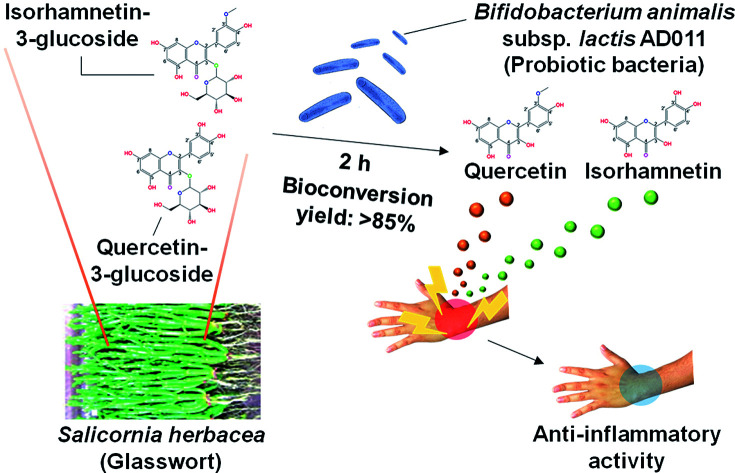

## Introduction

**Fig. 1:**
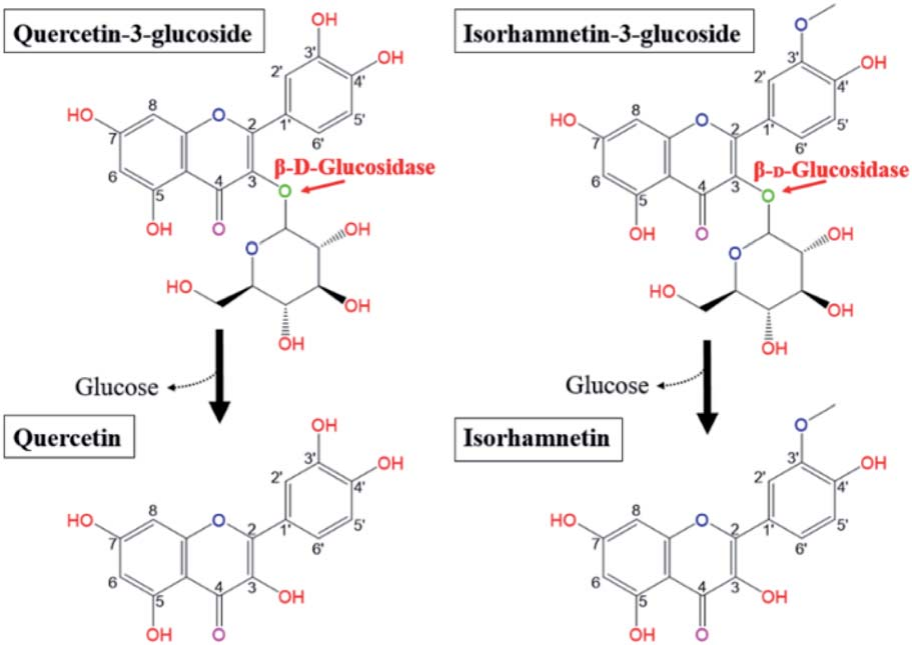
Biotransformation pathways from quercetin-3-glucoside and isorhamnetin-3-glucoside to quercetin and isorhamnetin by β-glucosidase, respectively.

*Salicornia herbacea* L., also known as glasswort, is widely distributed in saline soil areas such as the seashore, foreshore, and salt lakes in Korea, Japan, Iran, the United States, and European countries.^1–5^ As an herbal halophyte, *S. herbacea* has been used as a folk medicine in Asian countries for the treatment of constipation, diabetes, nephropathy, hepatitis, and diarrhea.^2,3,6^ Multiple scholars have reported that *S. herbacea* exhibits multiple bioactive functionalities, including antioxidant, anticancer, anti-inflammatory, osteoblastogenesis, anti-hyperglycemic, and anti-hyperlipidemic effects.^5–14^ Recently, *S. herbacea* has been widely cultivated and processed in various forms as commercially available functional cosmetics, dietary supplements and medicines due to its proven health benefits and marketing strengths.^1,3^ Previous phytochemical studies revealed that *S. herbacea* contains bioactive molecules such as flavonoids, minerals, and polysaccharides.^1,2,5,6,15–17^ Among them, flavonol glycosides, especially quercetin-3-glucoside (Q3G) and isorhamnetin-3-glucoside (IR3G), have been regarded as the principle substances responsible for the observed biofunctional activities.^18,19^ Q3G is a member of a group of flavonoids that have a glucose moiety at position 3; the aglycone form is known as quercetin (Q). Quercetin is a representative flavonol and has a 2-phenyl chromen-4-one backbone and a double bond between carbons 2 and 3 with five hydroxy groups at positions 3, 5, 7, 3′, 4′.^20^ Isorhamnetin has the same backbone as quercetin and isorhamnetin is the methylated form of quercetin at position 3′. IR3G has a glucose moiety in position 3 (Fig. 1).^21^

Research has shown that the specific makeup of each individual microbiome affects the range of catalysis occurring in each individual.^22^ Hasegawa^23^ reported that the pathways employed in *in vivo* conversion of glycosides may be different due to the diversity of microorganisms present in each host's gut. Therefore, orally consumed flavonoid molecules are catalysed and structurally transformed into deglycosylated flavonoid forms (aglycones) by gut microbiota and their glycosidases.^24^ Flavonoids in the form of aglycones are known to be more efficiently transferred into the bloodstream from the intestinal tract and more effectively act as bioactive molecules than the flavonol glycosides from which they are produced.^25–27^ Glucose is the most common sugar moiety of flavonol glycosides in plants, and the β-glucosidic bonds of Q3G and IR3G are possibly deconjugated *via* β-glucosidase catalysis.^24,28–31^ This catalysis would produce quercetin and isorhamnetin (Fig. 1). Certain microbial strains, such as *Bacteroides*,^32^*Clostridium*,^33,34^ and *Eubacterium*,^35,36^ are capable of structurally transforming flavonol glycosides into functional aglycones. However, many of these microbes are not suitable for food processing applications due to safety and marketing concerns. Also, many of the previously mentioned studies were limited to the biocatalysis of soybean isoflavones. The principle objective of this research was, therefore, to develop effective quercetin and isorhamnetin production using biotransformation of Q3G and IR3G in *S. herbacea via* probiotic enzyme catalysis. We screened potential probiotic strains, obtained β-glucosidase from selected cell strains, and evaluated their catalytic substrate transformation capabilities. After quercetin and isorhamnetin production, we conducted qualitative (liquid chromatography/mass spectrometry [LC/MS] and cell inflammatory assays) and quantitative (high-performance liquid chromatography [HPLC]) analyses to demonstrate the practicality of our method, which is potentially applicable to commercial quercetin and isorhamnetin production. Commercial production of quercetin and isorhamnetin *via* probiotic bacteria could lead to the development of treatments for inflammation-associated diseases.

## Experimental

### Extraction of quercetin-3-glucoside and isorhamnetin-3-glucoside from *S. herbacea*

*S. herbacea* powder was generously donated by Daeshinhamcho company (Sinan, Korea). *S. herbacea* was cultivated from March to July and harvested at the seashore of Sinan-gun in Korea. Harvested *S. herbacea* was heat-dried (60 °C, 30 h) or freeze-dried (−65 °C, *in vacuo*, 30 h), and ground. To extract Q3G and IR3G, water, 70% methanol, or 100% methanol were used as solvents. One hundred grams of *S. herbacea* powder was added to 1 L of each solvent and held at 80 °C in a water bath shaking at 120 rpm for 4 h. *S. herbacea* particles were filtered out using 0.45 μm microfilters. The collected permeates were evaporated and fractionated by dichloromethane, *n*-hexane, and *n*-butanol (1 L each with the same volume of water) to remove chlorophyll, lipids, proteins, and sugars.^18^ The insoluble residue of the *n*-butanol fraction was removed by filtration (Whatman® Grade 3, Z240478, Maidstone, England) and the collected filtrate was evaporated using an Eyela N-100 rotary evaporator (Rikakikai Co. Ltd., Tokyo, Japan) and dissolved in 500 mL methanol. A portion of the methanol suspended *n*-butanol residue was fractionated by loading 5 mL onto an activated SPE® Vac C18 column (Sep-Pak, WAT054945, Waters Corp, Milford, MA, USA) and eluting with a sequence of water, methanol/water 40–60% (v/v), and 100% methanol. The 100% methanol fraction (the flavonoid-enriched fraction) was evaporated and resuspended in water for biotransformation.

### Preparation of microbial crude enzyme and β-d-glucosidase assay

The following probiotic strains were generously donated by BIFIDO LTD (Hongcheon, Korea): *Lactobacillus delbrueckii* sp. *delbrueckii KCTC 1047* (LD1047), *Lactobacillus delbrueckii* subsp. *bulgaricus KCTC3188* (LD3188), *Lactobacillus cacei KCTC 3109* (LC3109), *Bifidobacterium adolescentis Int57* (Int57), *Leuconostoc paramesenteroides KCTC 3531* (LP3531), *Bifidobacterium* sp. *SJ32* (SJ32), *Bifidobacterium infantis KCTC 3249* (BI 3249), *Bifidobacterium* sp. *SH5* (SH5), *Bifidobacterium bifidum BGN4* (BGN4), and *Bifidobacterium animalis* subsp. *lactis AD011* (AD011). All bacteria were grown in Lactobacilli MRS medium (Becton-Dickinson Company, Detroit, MI, USA), supplemented with 0.05% (w/v) l-cysteine·HCl at 37 °C under anaerobic conditions for 18 h, and subcultured in 50 mL of MRS medium. Cultured probiotic cells were collected *via* centrifugation (3000 × *g* for 30 min at 4 °C). The collected cells (8 log CFU mL^−1^) were washed twice with 50 mM phosphate buffer (PB, pH 6.0) and resuspended in 10 mL of phosphate buffer. To separate the intracellular enzymes from the cytosol, all cells were disrupted *via* sonication (Sonics and Materials, Inc., Newtown, CT, USA) at 4 °C with 1 s burst pulse and 1 s cooling intervals at an amplitude of 45 for 30 min, as in our previous study.^31^

β-d-Glucosidase activity in the disrupted cell suspensions of all ten probiotic strains were assayed by the degradation of artificial substrates to produce free *p*-nitrophenols. *Para*-nitrophenyl-β-d-glucopyranoside (Sigma-Aldrich, #N7006, St. Louis, MO, USA) was used as an artificial substrate. Twenty μL of crude enzyme extract and 20 μL of 5 mM *p*-nitrophenyl-β-d-glucopyranoside were combined with 60 μL of PB (0.02 M, pH 6.0) in each well of a 96 well plate (SPL, #32296, Pocheon, Korea). The mixtures were incubated at 37 °C for 8 min, shaking at 100 rpm. The enzyme reaction was stopped by adding 100 μL of 0.5 M Na_2_CO_3_, and the released *p*-nitrophenol was measured with a 96 well microplate reader (Bio-Rad Laboratories, Philadelphia, PA, USA) at 405 nm. To obtain the specific activity, enzyme activity was divided by mg of protein. The level of protein was evaluated *via* a BCA protein Assay Kit (Pierce™, CAT 23225, Waltham, USA).

### Biotransformation of quercetin-3-glucoside and isorhamnetin-3-glucoside from *S. herbacea* using microbial crude enzyme extract and preparation of isorhamnetin-3-glucoside, quercetin-3-glucoside, isorhamnetin and quercetin

Biotransformation of Q3G and IR3G was conducted based on our previous study.^24,28–30,37–39^ Crude *S. herbacea* extracts were resuspended in a 60 μL of 50 mM PB (pH 6.0), mixed with 940 μL of microbial crude enzyme extract, and incubated at 37 °C in a water bath shaking at 150 rpm (0, 0.5, 1, 1.5, 2, 8, 16, 36 h). After biotransformation, mixtures were boiled (100 °C) for ten minutes to terminate enzyme reaction. Finally, mixtures were freeze-dried and resuspended in MeOH for analysis. The biotransformation percentage was calculated by the following formula:^37,40^Conversion percentage of quercetin (or isorhamnetin) content (%) = (transformed content of quercetin (or isorhamnetin) after biotransformation at given incubation time/(residual content of Q3G (or IR3G) + transformed content of quercetin (or isorhamnetin) after biotransformation at given incubation time)) × 100.

### Analysis of quercetin-3-glucoside, isorhamnetin-3-glucoside, quercetin, and isorhamnetin using chromatographic methods

Q3G, quercetin, and isorhamnetin standards were purchased from Sigma-Aldrich and an IR3G standard was purchased from Extrasynthese (Lyon, France). Stock solutions of each standard compound and lyophilized samples were dissolved in methanol, filtered through 0.45 μm syringe filters (Pall, Ann Arbor, MI, USA), and used for HPLC analysis. The separation and measurement of flavonoids were performed on a Dionex P680 HPLC (Dionex Corporation, Sunnyvale, CA, USA) equipped with an ASI-100 auto sampler (Dionex Corporation, Sunnyvale, CA, USA) and a UVD 170 UV-vis detector (Dionex Corporation, Sunnyvale, CA, USA). A Waters Sunfire C18 column (4.6 mm × 150 mm, 3.5 μm particle size, Waters Corporation, Milford, MA, USA) and a TCC-100 thermostatically controlled column compartment (Dionex Corporation, Sunnyvale, CA, USA) were used; the column was maintained at 30 °C during the analysis. The mobile phase consisted of solvent A (0.1% [v/v] trifluoroacetic acid [TFA] in water, pH 2.5) and solvent B (acetonitrile). The gradient program was: 0–5 min, 10% B; 5–45 min, linear gradient from 10% to 40% B. The injection volume of standards and samples was 20 μL, and the flow rate was 0.8 mL min^−1^. Simultaneous detection at 254 nm and 370 nm was accomplished.

Sample molecular weights were determined and compared with standard compounds using an Agilent 6410A triple quadrupole LC-MS system (Agilent Technologies, MA, USA) equipped with a Waters (Milford, MA, USA) Sunfire C18 column (150 mm × 4.6 mm, 3.5 μm particle size). Mass spectrometric analysis was carried out at the Central Laboratory for Instrumental Analysis at Kyung Hee University's Global Campus (Yongin, South Korea).

In order to obtain isorhamnetin and quercetin from *S. herbacea* extracts, enzyme-treated samples were subjected to enzyme inactivation *via* heat treatment for 10 min at 100 °C, followed by freeze-drying. The dried samples were dissolved in 100% methanol and used to separate isorhamnetin and quercetin fractions *via* semi-preparative HPLC. The preparative HPLC (Young Lin Acme 9000, Younglin Instrument Co., Ltd., Anyang, Korea), equipped with a semi-preparative ZORBOX SB-C18 5 μm, 9.4 mm × 250 mm column (Agilent Technologies, Santa Clara, CA, USA), was utilized. The mobile phase consisted of solvent A (0.1% (v/v) trifluoroacetic acid in HPLC grade water, pH 2.5) and solvent B (methanol) with the following gradient: 0–20 min, linear gradient from 65% to 75% B; 20–35 min, 75–80% B, 35–50 min, 80–100% B. The injection volume of the samples was 1 mL, the flow rate was 5 mL min^−1^, and the absorbance was measured using a UV detector (UV VIS detector, Younglin Instrument Co., Ltd., Anyang, Korea) at a wavelength of 254 and 370 nm. Q3G and IR3G were extracted by the same method as above but the *S. herbacea* extracts were not treated with microbial enzyme. Each collected Q3G, quercetin, IR3G, and isorhamnetin in HPLC solvent (water and methanol) were evaporated using an Eyela rotary evaporator N-100 (Rikakikai Co., Ltd., Tokyo, Japan) and used for evaluation of anti-inflammatory effects.

### Evaluation of anti-inflammatory effects of quercetin-3-glucoside, isorhamnetin-3-glucoside, quercetin, and isorhamnetin

The anti-inflammatory effects of Q3G, quercetin, IR3G, and isorhamnetin were evaluated by measuring the production of proinflammatory cytokines TNF-α and IL-6 in LPS-induced RAW 264.7 cell line cells using commercially available enzyme-linked immunosorbent assay (ELISA) kits. RAW 264.7 cells (KCLB 40071) were obtained from the Korean Cell Line Bank (Seoul, Korea). These cells were maintained and subcultured according to the distributor's instruction. Briefly, these cells were cultured in Dulbecco's modified Eagle's medium (GIBCO, 12491-023, Carlsbad, CA, USA) with 10% (v/v) fetal bovine serum (GIBCO, 12483-020, Carlsbad, CA, USA) and 1% (v/v) antibiotic-antimycotic solution (GIBCO, R25005, Carlsbad, CA, USA) at 37 °C in a humidified atmosphere of 95% air and 5% CO_2_.^37^

To evaluate anti-inflammatory effects, RAW 264.7 cells were seeded at 1 × 10^4^ cells per well in a 96-well plate and incubated at 37 °C for 22 h. The cells were then treated and incubated with 1, 5, or 10 μM of Q3G, quercetin, IR3G, or isorhamnetin for 2 h. LPS (0.1 μg mL^−1^) was added to each cell in the 96-well plate and the plate again incubated for 24 h. After incubation, the levels of TNF-α and IL-6 in 100 μL of each cell supernatant were measured using ELISA kits (BD OptEIA™ Mouse TNF ELISA Kit, 560478, BD Pharmingen, San Diego, Calif., USA and BD OptEIA™ Mouse IL-6 ELISA Kit, 550950, BD Pharmingen, San Diego, Calif., USA) according to the manufacturer's protocols.

The cytotoxicity of Q3G, quercetin, IR3G, and isorhamnetin was evaluated by MTT assay. In brief, RAW 264.7 cells were seeded at 5 × 10^4^ cells per well in a 96-well plate (Corning® 96 Well, #3596, Corning, NY, USA) and incubated at 37 °C for 22 h. Either 1, 5, or 10 μM of Q3G, quercetin, IR3G or isorhamnetin was added to each cell and the plate was again incubated for 2 h. LPS (0.1 μg mL^−1^) (Sigma-Aldrich, L4516, St. Louis, MO, USA) was added to each of the 96 cells and the plate was incubated for 24 h. After this incubation, a 10% (v/v) MTT stock solution (5 mg mL^−1^) was added to each well, followed by incubation at 37 °C for 2 h. After centrifugation at 100*g* for 5 min at 4 °C, the supernatants were removed. The converted formazan product was dissolved in 200 μL of dimethyl sulfoxide (DMSO) and the absorbance was measured at 540 nm using a microplate reader (Bio Rad Laboratories, Inc., Hercules, CA, USA). The percentage of viable cells was estimated compared with that of the untreated control cells.

## Results and discussion

### The effect of drying methods and extraction conditions on the quantity of quercetin-3-glucoside and isorhamnetin-3-glucoside extracted from *S. herbacea*

Several kinds of flavonoids (IR3G,^19^ Q3G,^18^ 2*S*-2′,7-dihydroxy-6-methoxyflavanone, 2*S*-2′-hydroxy-6,7-dimethoxy-flavanone, and 2*S*-5,2′-dihydroxy-6,7-methylenedioxyflavanone^2^) have been reported to exist in *S. herbacea*, and these flavonoids have been separated and identified using spectroscopic methods such as HPLC, MS, and NMR. Among them, Q3G and IR3G have been regarded as the key bioactive molecules of *S. herbacea*.^18,19^ However, the bioavailabilities of these two flavonoid substances vary *in vivo* depending on the presence of a sugar residue. Flavonol aglycones do not have sugar residues and are known to have better functional properties than those of flavonol glycosides.^41,42^ Extraction and separation protocols for these flavonol glycosides are not yet standardized. Because the major goal of our study was to obtain and assess biotransformed products (quercetin and isorhamnetin) *via* enzymatic catalysis, we needed to develop effective extraction and separation protocols of mother molecules (Q3G and IR3G, respectively) in *S. herbacea*. We therefore compared the efficiency of using a combination of two drying methods (heat and freeze-drying) and three solvents (water, 70% MeOH and 100% MeOH) for extraction. All extracted molecules were quantitatively and qualitatively analysed by HPLC and LC/MS.

Both quercetin and isorhamnetin have flavonoid structures in which two phenyl groups are structurally linked by three carbon bridges which form an aromatic ring in a closed structure, and both molecules have double bonds on carbon numbers 2 and 3. The aromatic part of flavonol molecules show hydrophobic properties. However, the flavonols, quercetin and isorhamnetin, also have hydroxyl groups at 3, 5, 7, 3′, 4′ and exhibit hydrophilic properties. Q3G is the conjugated form of carbon number 3 of quercetin and carbon number 1 of glucose with a β-glycosidic linkage. The presence of glucose in Q3G conferred more hydrophilic properties compared to quercetin. Isorhamnetin has a flavonol backbone structurally similar to quercetin. However, isorhamnetin, unlike quercetin, has a methyl group instead of OH at the 3′ carbon of the aromatic ring and is therefore slightly more hydrophobic than quercetin. The glucose molecule in IR3G is also linked *via* a β-glucoside bond, which makes it slightly more hydrophilic than isorhamnetin. Traditionally, various organic solvents (*e.g.* methanol, ethanol, butanol and chloroform) or water have been used when extracting flavonoids from plants. Among these organic solvents, methanol has a polarity of 6.6, which is known to be higher than the other organic solvents and possibly has a high affinity with quercetin or isorhamnetin. Due to the structural properties of Q3G and IR3G with their low-polarity, organic solvents or aqueous-based methanol solutions are normally used for their extraction from plant materials.^43,44^ Both quercetin-3-glucose and isorhamnetin-3-glucose have a glucose moiety with hydrophilic properties in common. Therefore, we used methanol and aqueous-based solvents (water and 70% methanol) to extract quercetin-3-glucose and isorhamnetin-3-glucose from *S. herbacea*.

As a result, the extraction efficiency of Q3G using methanol as a solvent was 3.9–4.2 times higher than that using water as a solvent and 1.1–1.2 times higher than using 70% methanol as a solvent. Additionally, the extraction efficiency of IR3G using methanol was 6.5–6.7 times higher than with water extraction and 1.2 times higher than 70% methanol extraction (Table 1).

**Table 1:** Comparison of quercetin-3-glucoside and IR3G content of *S. herbacea* extract after heat-drying, and freeze-drying, using different solvents^a^

Contents	Molecules	Drying methods	Extraction solution		
			Water	70% methanol	100% methanol
Concentration (μg mL^−1^)	Q3G^b^	Heat-drying	10.4 ± 0.31^c^	33.1 ± 0.8^b^	40.5 ± 1.4^a^
		Freeze-drying	14.3 ± 0.2^c^	56.6 ± 0.8^b^	60.4 ± 0.8^a^
	IR3G^c^	Heat-drying	7.8 ± 0.0^c^	42.7 ± 0.0^b^	52.0 ± 0.3^a^
		Freeze-drying	15.4 ± 0.3^c^	81.0 ± 1.1^b^	100.0 ± 2.0^a^

a Extractions were replicated three times and all values are presented as mean ± SD (*n* = 3). Different superscripts within the same rows indicate that values are significantly different at *p* < 0.05 (Tukey HSD and Games-Howell tests).

b Q3G denotes quercetin-3-glucoside.

c IR3G denotes isorhamnetin-3-glucoside.

The drying of *S. herbacea* before extraction is the most important step for increasing the yield of Q3G and IR3G. Traditionally, Korean people have used sunlight to dry a variety of vegetables and plant medicines (*e.g.* red peppers, radish and ginseng). For example, the quality and price of sun-dried red pepper is higher than red pepper dried *via* other techniques. Many Korean food companies market red pepper and red pepper-containing products such as kimchi and pepper paste using the term, “sun-dried” to highlight this processing technique. However, it has been reported that when plant flavonoids are exposed to UV radiation, their physical structures change.^45^ Flavonoids may also be structurally changed by enzymes in plant cells, endophytic microorganisms, high temperatures, and oxidative stress during the drying process. Therefore, natural drying is not the best technique for the preservation of flavonoid content in natural foods.^46–49^ To compare the quantity of Q3G and IR3G in *S. herbacea* after drying by heat or freeze-drying, samples were dried using both methods and extraction of the target flavonoids was executed using the previously described technique (water, 70% methanol and methanol at 80 °C for 4 h incubation). The quantity of Q3G remaining after freeze-drying was about 1.5 times higher than the quantity of Q3G remaining after heat-drying. Similarly, the quantity of IR3G after freeze-drying was about 1.9 times higher than the quantity of IR3G remaining after heat-drying. In light of these observations and using this general strategy, the effective Q3G and IR3G extraction from *S. herbacea* may be optimized based on the relative polarities of target compounds.

### β-Glucosidase screening from probiotic microorganisms

Probiotics have been widely used to produce glycosidases for transforming certain phytochemical glycosides, including ginsenosides, isoflavones, anthocyanins, and flavonols. Probiotic enzymatic biotransformation is widely used to transform phytochemicals because of the many advantages it offers, including low cost, easy reaction control and stereospecificity *vs.* the results generated using chemical or thermal processing techniques.^24,28–30,50,51^ Highly region-specific enzymatic transformations may be promising, but preparing for the purification of one specific enzyme is expensive in terms of time and money. To save both time and cost, crude enzyme extracts or fermentation may be used. Although these methods have significant advantages in creating target substances, the transformation should be carefully controlled since several enzymes exist in crude enzyme extracts.^20^ Crude enzyme extracts can also include enzymes that degrade target substance(s) or create other forms of the substance(s). Finally, if microorganisms are used to supply the enzymes, suitable specific organisms must be identified to accomplish the desired end product(s).^52,53^

**Fig. 2:**
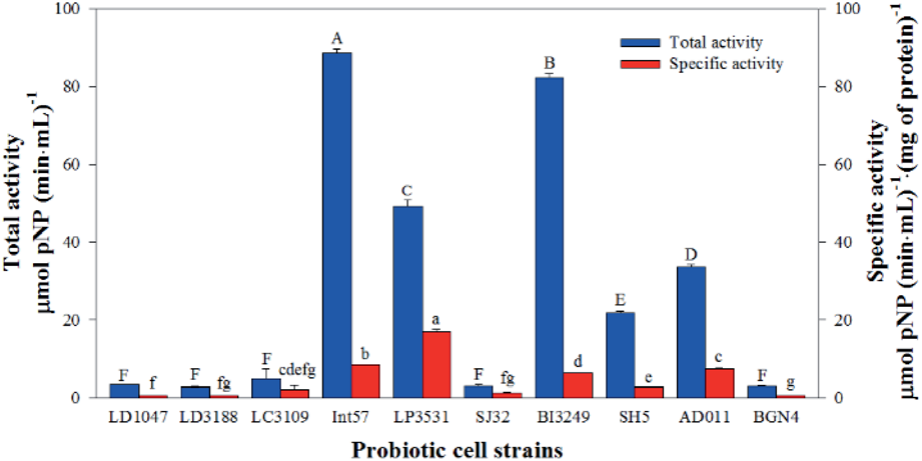
Total and specific β-d-glucosidase activities of 10 probiotic cell strains. One-way ANOVA followed by Games-Howell *post hoc* test was performed. Treatments with different letters are significantly different at *p* < 0.05 (*n* = 3).

In order to find a microorganism suitable for providing the enzymes required to transform Q3G and IR3G, we screened 10 food-grade microorganisms and evaluated their β-glucosidase activities based on their ability to cleave *p*-nitrophenyl-β-d-glucopyranoside into *p*-nitrophenol and glucose. *p*-Nitrophenol (*p*NP), an artificial substrate, can be enumerated by detection at 450 nm, allowing for a measurement of β-glucosidase activity (Fig. 2).

The total activities of the β-d-glucosidase within each crude enzyme extract ranged from 2.8 ± 0.5 to 88.4 ± 1.1 μmol *p*NP (min mL)^−1^, and the specific activities of the β-d-glucosidase within each crude enzyme extract ranged from 0.5 ± 0.0 to 17.0 ± 0.6 μmol *p*NP (min mL)^−1^ (mg of protein)^−1^. The total β-d-glucosidase activities of five probiotic extracts (LD1047, LD3188, LC3109, SJ32, and BGN4) were shown to be less than 5 μmol *p*NP (min mL)^−1^, which is significantly lower than other groups. The total β-d-glucosidase activities of the other five probiotic strains (Int57, LP3531, BI3249, SH5, and AD011) ranged from 21.7 ± 0.5 to 88.4 ± 1.1 μmol *p*NP (min mL)^−1^, with β-glucosidase activity levels higher than those of the other five probiotics. Thus, crude enzyme extracts of Int57, LP3531, BI3249, SH5, and AD011 were selected to transform Q3G and IR3G from *S. herbacea*.

### Biotransformation of quercetin-3-glucoside and isorhamnetin-3-glucoside into quercetin and isorhamnetin

Qualitative analysis *via* HPLC was performed to evaluate the conversion percentage of natural substrates (Q3G and IR3G) during biotransformation by β-glucosidase from Int57, LP3531, BI3249, SH5, AD011. Specifically, the overall level of substrate and end product were enumerated so we could measure the amount of substrate remaining that could be biotransformed to aglycones. When the conversion percentage was predicted using artificial substrate (*p*NP), AD011 showed a conversion percentage that ranked 4^th^ among the 5 strains. In the experiment with natural substrates, AD011 showed the highest glycoside catalytic activities against natural substrates among the five selected microbial strains (Table 2).

**Table 2:** The residual flavonol glycoside (Q3G [quercetin-3-glucoside] and IR3G [isorhamnetin-3-glucoside]) and aglycone (Q [quercetin] and IR [isorhamnetin]) contents after 8 h of reaction with crude extracts from five lactic acid bacteria strains^a^

No.	Molecules (μM)	Microorganisms					
		Control	Int57	LP3531	BI3249	SH5	AD011
1	Q3G^b^	107.8 ± 0.9^a^	17.7 ± 4.0^d^	31.8 ± 3.4^c^	47.6 ± 6.9^b^	38.4 ± 5.4^bc^	13.3 ± 4.3^d^
2	Q^c^	5.8 ± 1.0^d^	42.5 ± 0.9^b^	6.3 ± 0.3^d^	5.0 ± 0.9^d^	33.5 ± 2.6^c^	98.6 ± 3.1^a^
3	Total (sum.)	113.7 ± 1.9^a^	60.3 ± 4.9^bc^	38.1 ± 3.4^d^	52.6 ± 7.1^c^	71.9 ± 7.9^b^	112.0 ± 3.4^a^
4	IR3G^d^	179.2 ± 1.2^a^	6.7 ± 1.1^d^	58.2 ± 5.5^c^	86.1 ± 5.4^b^	68.9 ± 6.9^bc^	12.3 ± 3.2^d^
5	IR^e^	1.1 ± 0.0^e^	84.9 ± 4.1^b^	1.2 ± 0.0^e^	3.4 ± 0.0^d^	47.0 ± 2.9^c^	168.5 ± 0.6^a^
6	Total (sum.)	180.2 ± 1.2^a^	91.6 ± 5.1^b^	59.5 ± 5.6^c^	89.5 ± 5.4^b^	115.9 ± 9.8^b^	180.8 ± 2.7^a^

a Values with different superscripts within the same rows are significantly different at *p* < 0.05 (Tukey HSD and Games-Howell tests) and mean ± SD (*n* = 3).

b Q3G denotes quercetin-3-glucoside.

c IR3G denotes isorhamnetin-3-glucoside.

d Q denotes quercetin.

e IR denotes isorhamnetin.

AD011 transformed 88.4 ± 1.1% of Q3G to quercetin. The conversion percentage from the other four samples ranged from 4.5 ± 0.8 to 38.1 ± 0.8%. Also, the transformation percentage of IR3G to isorhamnetin after 8 h was 93.3 ± 0.4% when the same crude enzymes extracted from AD011 were applied, while the transformation percentage of IR3G from the other four samples ranged from 0.7 ± 0.0 to 47.0 ± 2.3% (*n* = 3) after 8 h incubation. In the case of Int57 and BI3249, the total β-glucosidase activities of these microorganisms against *p*NP were more than 2 times higher than the total β-glucosidase activities of AD011. However, the transformation percentages of Q3G and IR3G of Int57 and BI3249 were significantly lower than that of AD011. Although *p*-nitrophenol, quercetin, and isorhamnetin are structurally homologous (they are all linked to glucose by β-1,4 glucosidic linkages), different conformations of substrates might affect binding times and/or activation energies could be different.

When *S. herbacea* extracts were treated with crude enzymes from Int57, LP3531, BI3249 and SH5, the overall level of flavonol molecules (*i.e.* Q3G, quercetin, IR3G and isorhamnetin) were significantly decreased. However, quantitative assessment after bioconversion showed that the total amount of Q3G and quercetin in the AD011-administered group was not statistically different from the total Q3G and quercetin amount in the control group in which no microorganism was administered. The total amount of IR3G and isorhamnetin in the AD011-administered group was also not statistically changed after biotransformation process. The LP3531 treatment group showed the highest degradation percentage during transformation of Q3G to quercetin and IR3G to isorhamnetin, with 66.5 ± 2.5% (*n* = 3) and 67.1 ± 3.2% (*n* = 3), respectively. Int57, BI3249 and SH5 treated groups showed 36.8 ± 6.0 to 53.8 ± 5.5% (*n* = 3) degradation percentage for Q3G transformation and 35.7 ± 5.5 to 50.3 ± 3.0% (*n* = 3) degradation percentage for IR3G transformation. It is apparent that the crude enzyme from AD011 selectively transformed Q3G and IR3G into quercetin and isorhamnetin, respectively. However, other enzymes from the other four microorganisms may transform and degrade all four *S. herbacea* flavonoids into other compounds nonspecifically by modifying their backbones or functional groups.^54^

### Evaluation of *Bifidobacterium animalis* subsp. *lactis* AD011 biotransformation properties

When producing target molecules *via* biotransformation, not only the yield of the target molecule but also the conversion time should be considered. Guan *et al.*^55^ reported that when a crude enzyme extract of *Rhodopseudomonas palustris* was utilized for rutin conversion, the conversion percentage was only 2.68% and 81.09% after 5 and 21 h of catalytic reaction, respectively. According to Quan *et al.*,^56^ 12 h of *Microbacterium esteraromaticum*-derived β-glycosidase incubation was applied to remove the glucose moiety of ginsenoside Rb2 and generate compound Y. Compound K was then sequentially generated by further removing the arabinose moiety of compound Y. Specifically, 0.74 mg mL^−1^ of Rb2 was converted to 0.27 mg mL^−1^ and 0.1 mg mL^−1^ of compound Y and compound K (52.2% and 23.5% of molar conversion yield), respectively. Our β-glucosidase screening results indicate that crude enzyme preparations from AD011 produced the best biotransformation percentages and the lowest degradation percentages for transforming Q3G and IR3G (Table 2). Crude enzyme extracts of AD011 were therefore chosen for further experimentation over longer exposure times. Before and after transformation, Q3G, IR3G, quercetin, and isorhamnetin were quantified by HPLC and qualified by LC/MS in the crude enzyme extracts using Electrospray Ionization (ESI)-Triple Quadrupole LC/MS. A total of 89.4 ± 0.8% of Q3G was converted into quercetin and 92.7 ± 1.4% of isorhamnetin-3-glucoside was converted into isorhamnetin in 8 h with a crude enzyme extract of AD011.

When producing minor aglycones from herb extracts *via* microbial biotransformation processes, it is important to develop effective protocols to generate the target molecule(s) without generating unwanted by-products, which complicate downstream processing. Crude enzymes can be defined as complex organic mixtures containing enzymes and other cellular materials produced *via* cell/microbial lysis and breakage. When flavonoid molecules are biotransformed using crude enzyme extracts from bacteria, these unfractionated organic complexes may contain key enzymes that are essential for flavonoid bioconversion. However, some molecules in bacterial lysate can act as enzyme inhibitors and/or inhibitor producers *via* glycosylation, oxidation, sulphation, methylation, hydroxylation, and aromatic ring degradation due to the lack of enzyme purification.^54^ From the producers' point of view, these unintended chemical reactions can contribute to the production of unwanted by-products, leading to increased production costs, reduced target product recovery, and difficult downstream processing.

For example, Xu *et al.*^57^ attempted to produce Q3G through the microbial biotransformation of quercetin with *Gliocladium deliquescens* NRRL 1086 and produced their target substance *via* 3-*O*-glycosylation. However, they reported that when the glycosylation reaction occurred, an oxidation cleavage reaction of the C-ring occurred simultaneously. As a result, unwanted by-products (*e.g.* 2-protocatechuoly-phloroglucinol carboxylic acid) were generated in addition to Q3G, and 2-protocatechuoly-phloroglucinol carboxylic acids were chemically decomposed into 2,4,6-trihydroxybenzoic acid and protocatechuic acid after biocatalysis. Because of these additional reactions, they were only able to achieve 46% of the possible Q3G after 12 h of reaction. According to Krishnamurty *et al.*,^58^ rutin, a precursor of quercetin, was successfully converted to quercetin by detachment of glucose and rhamnose molecules in rutin by *Butryrivibrio* sp. crude enzymes. However, in their research quercetin was further degraded to phloroglucinol, CO_2_, 3,4-dihydroxybenzaldehyde due to additional enzyme reaction. Oka and Simpson^59^ also reported that multiple unwanted by products, including carbon dioxide and 2-protocatechuoly-phloroglucinol carboxylic acid, are produced by enzymatic oxygenation and quercetinase in quercetin biosynthesis using *Aspergillus flavus*. According to Schneider *et al.*,^35^ growing *Clostridium orbiscindens* degraded 0.5 mM quercetin and structurally converted it to 3,4-dihydroxyphenylacetic acid in 6 h. Braune *et al.*^60^ reported that *Eubacterium ramulus* and its enzymes converted quercetin to 3,4-dihydroxyphenylacetic acid through taxifolin and alphitonin as intermediates with the reduction of the double bond at the 2,3-position and C-ring fission. In order to avoid unwanted enzyme reactions with flavonoid degradation and maximize quercetin and isorhamnetin productivities, proper enzyme host selection is crucial. Instead of host selection, separation of enzymes or proteins which catalyze unwanted reactions can be conducted, but purification of specific enzymes is a time consuming and expensive process. Therefore, using less purified crude enzyme extracts that are more appropriate for the specific biotransformation desired is a better choice.

**Fig. 3:**
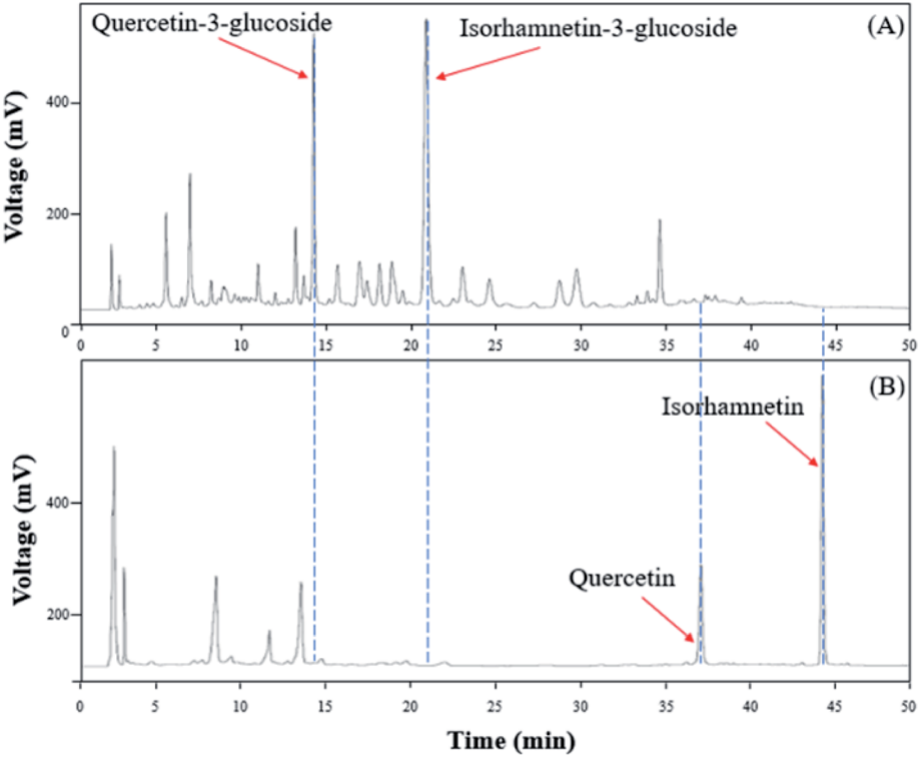
HPLC chromatogram showing changes in molecular distribution of *S. herbacea* extracts before and after bioconversion using crude AD011 enzyme.

**Fig. 4:**
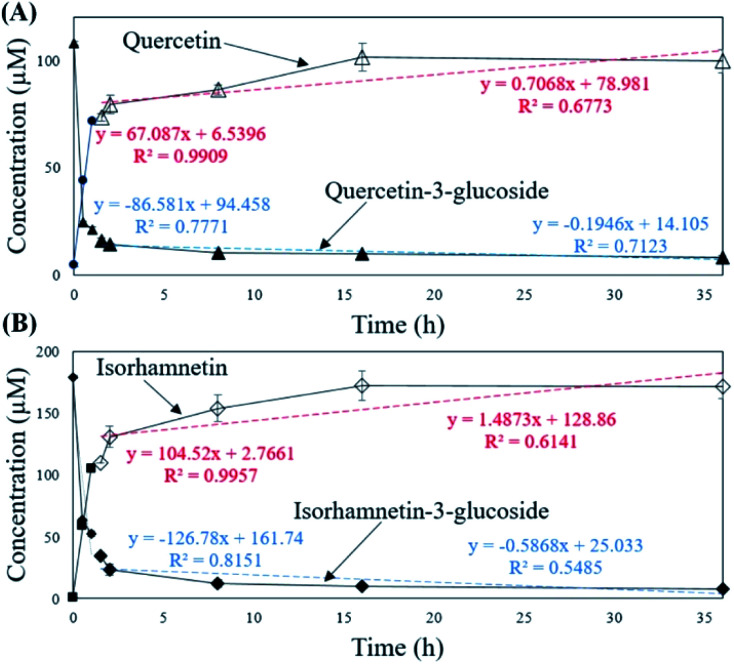
Concentrations of (A) quercetin-3-glucoside (▲) and quercetin (△) and (B) isorhamnetin-3-glucoside (◆) and isorhamnetin (◇) in *S. herbacea* extracts during 36 h incubation with crude AD011 β-glucosidase.

To verify that there was no structural change in the aglycone products after exposure to the AD011 crude enzyme over an extended time, the transformation reaction was conducted for 36 h. After about 16 h, Q3G and IR3G were converted to quercetin and isorhamnetin at percentage of 91.0 ± 0.8% and 94.8 ± 0.4%, respectively (Fig. 3 and 4). The transformed quercetin and isorhamnetin molecules had no further metabolization or transformation; Q3G reached 92.6 ± 0.4% and IR3G reached 95.5 ± 0.4% after 36 h. No structural degradation of the four flavonoids (Q3G, IR3G, quercetin, and isorhamnetin) was observed during this time. Enzyme exposure was extended an additional 20 h under the same conditions and again, no significant changes to the flavonoids and no degradation products were observed.

**Fig. 5:**
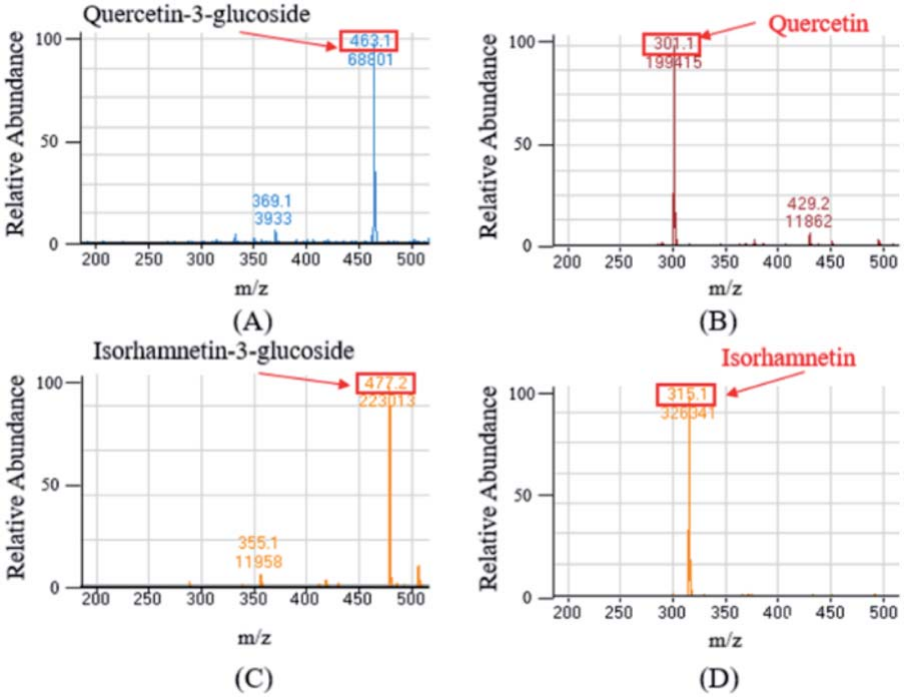
Mass spectrum of quercetin-3-glucoside (A) and isorhamnetin-3-glucoside (C) from *S. herbacea* and their transformed aglycones, quercetin (B) and isorhamnetin (D).

The ESI-MS were performed in the negative mode to determine the molecular weights of the compounds of interest. In the negative ion mode, the deprotonated ion [M–H]^−^ of Q3G, IR3G, quercetin, and isorhamnetin was at *m*/*z* 463.1, 477.2, 301.1, 315.1, displaying a sharp and distinguished peak. These results confirm that the crude enzyme extract of AD011 successfully transformed Q3G and IR3G into quercetin and isorhamnetin with enzymatic hydrolysis of the β-1,4-glycosidic linkage without further unwanted reactions (Fig. 5).

### The anti-inflammatory effects of quercetin and isorhamnetin compared to quercetin-3-glucoside and isorhamnetin-3-glucoside on LPS-induced TNF-α and IL-6 production in RAW 264.7 mouse macrophages

The anti-inflammatory effects of *S. herbacea* are reported to be due to the IR3G present. The anti-inflammatory effect is a result of IR3G suppressing lipopolysaccharide (LPS)-induced nitric oxide production, inducible nitric oxide synthase (iNOS), tumor necrosis factor-α (TNF-α), and interleukin-1β (IL-1β) in Raw 264.7 cells.^61^ Lipopolysaccharide (LPS) in the outer membrane of Gram-negative bacteria is recognized by the toll-like receptor 4 (TLR4) of the macrophage, which in turn activates macrophages to serve as innate immune cells. TLR4 signaling causes macrophage inflammation signal transduction, which translocates the transcription factor NF-κB in the cytosol into the nucleus, resulting in the expression of macrophage inflammatory genes. NF-κB and AP-1 are transcriptional regulators that regulate the expression of inflammatory mediators and cytokines at the transcription level. LPS artificially added to the RAW 264.7 cell medium stimulates macrophages to produce inflammatory mediators (*e.g.* inducible nitric oxide synthase [iNOS] and COX-2) or cytokines (*e.g.* TNF-α, IL-6, and interleukin [IL]-1β) as the result of intrinsic immune responses at high concentrations. These inflammatory responses play an important role in the innate immunity of the human body, but excessive production of inflammatory cytokines, such as TNF-α, IL-6, and IL-1β, may cause systemic inflammatory response disease syndrome (SIRS), severe tissue damage and/or sepsis. When septic shock occurs in the human body, it causes multiple organ (*e.g.* kidney, liver, and lung) failure and lethality rates are high. In addition, chronic inflammation has been reported to be associated with cancer, neurological diseases, heart disease, stroke, rheumatoid arthritis, and atopic dermatitis. Flavanoids and their subclasses, flavanones, flavones, flavonols, anthocyanidins, and isoflavones are known to have nutraceutical activities, including anticancer, anti-inflammatory, antiviral, and antimicrobial effects. Various groups have demonstrated that flavonol molecules have anti-inflammatory capacities through *in vitro*, *in vivo* and clinical research (Table 3).

**Table 3:** Anti-inflammatory effects of quercetin derivatives

Compounds	Concentration	Cell lines	Induced by	Inhibited inflammatory mediators	Ref.
Quercetin	1–30 μM	Mouse BV-2 microglia	LPS/IFN-γ	NO^a^, iNOS^b^ (mRNA), IKK^c^, NF-κB^d^, STAT1^e^, AP-1^f^	61
Quercetin-3-sulfate	10 μM			NO^a^ (not inhibited)	
Quercetin	1–10 μM	Mouse BV-2 microglia	LPS/IFN-γ	NO^a^, phosphorylation of ERK^g^, JNK^h^, p38^i^, Akt^j^, JAK-1^k^, Tyk2^l^, and Src^m^	62
Quercetin	1–50 μM	RAW 264.7	LPS	NO^a^, TNF-α^u^	63
Quercetin	100 μM	Rat peritoneal macrophages	LPS	NO^a^, phosphorylation of p44/42 MAPK, p38^i^ MAPK^n^, JNK^h^	64
Quercetin-3-glucoside					
Hyperin					
Quercetin	16–500 μM	RAW 264.7	LPS/IFN-γ	NO^a^, TNF-α^u^	65
Quercetin-3-glucoside				TNF-α^u^ (not inhibited)	
Isorhamnetin	12.5–50 μM	RAW 264.7	LPS	Ho-1^o^ mRNA expression, IL-6^p^, NF-κB^d^ p50, STAT1^e^	66
Quercetin	Different concentrations	Human platelets	Calcium ionophore	12-HHT^q^, TXB2^r^, PGE2^s^, 12-HETE^t^	41
Isorhamnetin					
Isorhamnetin-3-glucoside					
Isorhamnetin-3-glucoside	0.1–10 μg mL^−1^ (0.2–20 μM)	RAW 264.7	LPS	iNOS^b^ (protein level), TNF-α^u^, IL-1β^v^	9

a NO, nitric oxide.

b iNOS, inducible nitric oxide synthase.

c IKK, IκB kinase.

d NF-κB, nuclear factor-kappa B.

e STAT1, signal transducer and activator of transcription-1.

f AP-1, activating protein-1.

g ERK, extracellular signal-regulated kinase.

h JNK, c-Jun N-terminal kinase.

i p38, mitogen-activated protein kinase p38.

j Akt, protein kinase B.

k JAK-1, Janus kinase-1.

l Tyk2, tyrosine kinase 2.

m Src, proto-oncogene tyrosine-protein kinase.

n MAPK, mitogen-activated protein kinase.

o Ho-1, heme oxygenase-1.

p IL-6, interleukin 6.

q 12-HHT, 12(*S*)-hydroxy(5*Z*,8*E*,10*E*)-heptadecatrienoic acid.

r TXB2, thromboxane B2.

s PGE2, prostaglandin E2.

t 12-HETE, 12(*S*)-hydroxy-(5*Z*,8*Z*,10*E*,14*Z*)-eicosatetraenoic acid.

u TNF-α, tumor necrosis factor alpha.

v IL-1β, interleukin 1 beta.

It has been reported that quercetin inhibits the activation of IKK, NF-κB, STAT1, iNOS, NO, TNFα, MAPKs, Akt, Src, JAK-1 and Tyk2. Inhibition of nuclear translocation of NF-κB p65 is known to inhibit expression of IL-1β, TNF-α and IL-6. Specifically, quercetin inhibits the expression of IL-1β, TNF-α and IL-6 by inhibition of nuclear translocation of NF-κB p65.

Manjeet and Ghosh^63^ reported that the production of LPS-induced nitric oxide and tumor necrosis factor-α (TNF-α) from macrophage RAW 264.7 cells was inhibited by 1–50 μM of quercetin in a dose-dependent manner. They also used L929 cells to demonstrate that the level of TNF-α produced by Raw 264.7 cells is reduced by quercetin. L929 cells are known to induce apoptosis by TNF-α. In their work, L929 cells were cultured after quercetin and LPS were administered to Raw 264.7 cells. As a result, 50 μM quercetin inhibited the apoptosis of L929 cells by >80%. Endale *et al.*^67^ reported that quercetin blocked Src and Syk associated with PI3k, PDK1, and AKT activation and blocked the association of P85 and TLR4/MyD88, resulting in inhibition of downstream signalling pathways IRAK1, TRAF6, activation of TAK and IKKα/NF-κB phosphorylation. These intracellular responses reduce the secretion and mRNA expression of the proinflammatory cytokines TNF-α and IL-6.

Lee, *et al.*^64^ extracted quercetin, Q3G, and hyperin (forms with galactose at quercetin carbon 3) from *Acanthopanax chiisanensis*, which has traditionally been used for the treatment of inflammation in Asia. They studied whether these three substances inhibit the nitrite production of LPS-induced rat peritoneal macrophages. In their work, the inhibition percentage of quercetin was 66.1% at a concentration of 100 μM. In addition, quercetin inhibited the phosphorylation of pp44/42 MAPK, p38 MAPK and JNK compared to the other two substances. Wang *et al.*^65^ also tested whether anthocyanins and flavonoids inhibit TNF-α production of RAW 264.7 activated with LPS and IFN-γ. Quercetin was shown to inhibit TNF-α production in a concentration-dependent manner at 125, 250 and 500 μM. Isorhamnetin is a 3′-methoxylated derivative of quercetin. Jin *et al.*^66^ confirmed that IL-6 production of LPS-induced RAW 264.7 was decreased in a dose-dependent manner by administration of 12.5, 25, 50 μM isorhamnetin at mRNA and protein levels. Lesjak *et al.*^41^ evaluated the IC_50_s of quercetin, isorhamnetin, IR3G that can reduce amount of inflammation mediators derived from arachidonic acid (*e.g.* 12(*S*)-hydroxy(5*Z*,8*E*,10*E*)-heptadecatrienoic acid (12-HHT), thromboxane B2 (TXB2), and 12(*S*)-hydroxy-(5*Z*,8*Z*,10*E*,14*Z*)-eicosatetraenoic acid (12-HETE) from human platelets). Among the three flavonoids, quercetin showed the highest anti-inflammatory activity, followed by isorhamnetin and IR3G. In the case of IR3G, Kim *et al.*^9^ confirmed that IR3G extracted from *S. herbacea* inhibited NO, iNOS, TNF-α, and IL-1β production in LPS-induced RAW 264.7. However, they did not study the inflammation inhibitory effect of isorhamnetin.

**Fig. 6:**
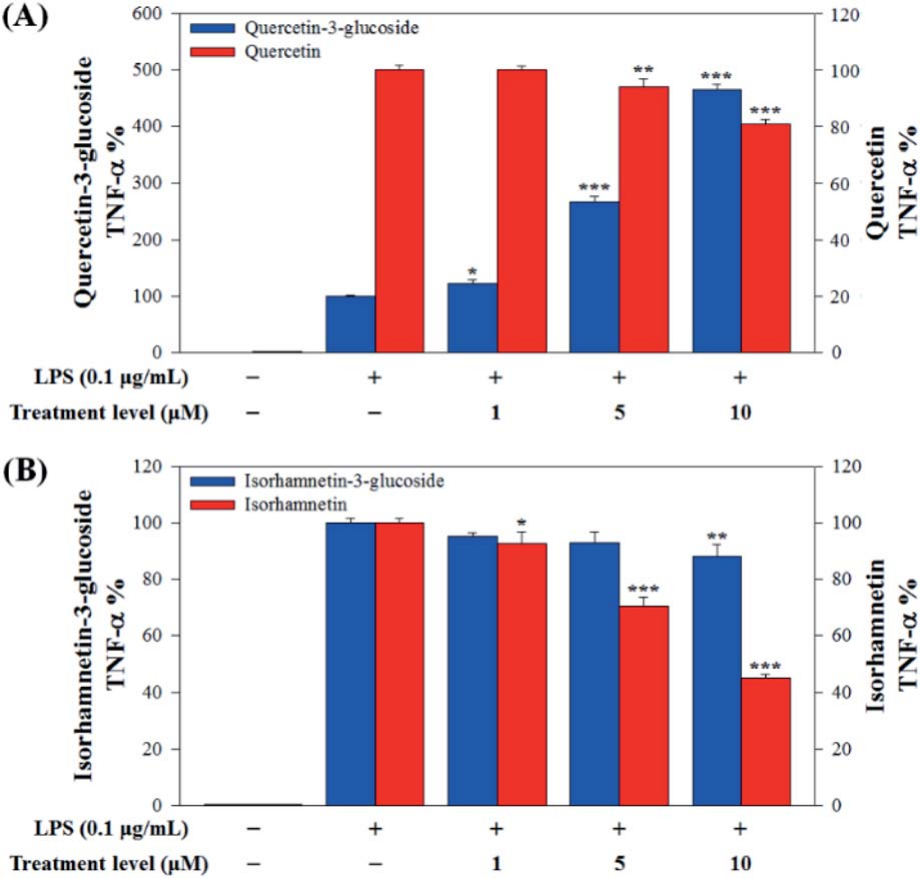
(A) Comparison of TNF-α production levels with quercetin-3-glucoside and quercetin and (B) isorhamnetin-3-glucoside and isorhamnetin. Values are the mean ± SD of three independent experiments. (*) *p* < 0.05, (**) *p* < 0.01, and (***) *p* < 0.001 indicate significant differences compared to the LPS-treated group.

**Fig. 7:**
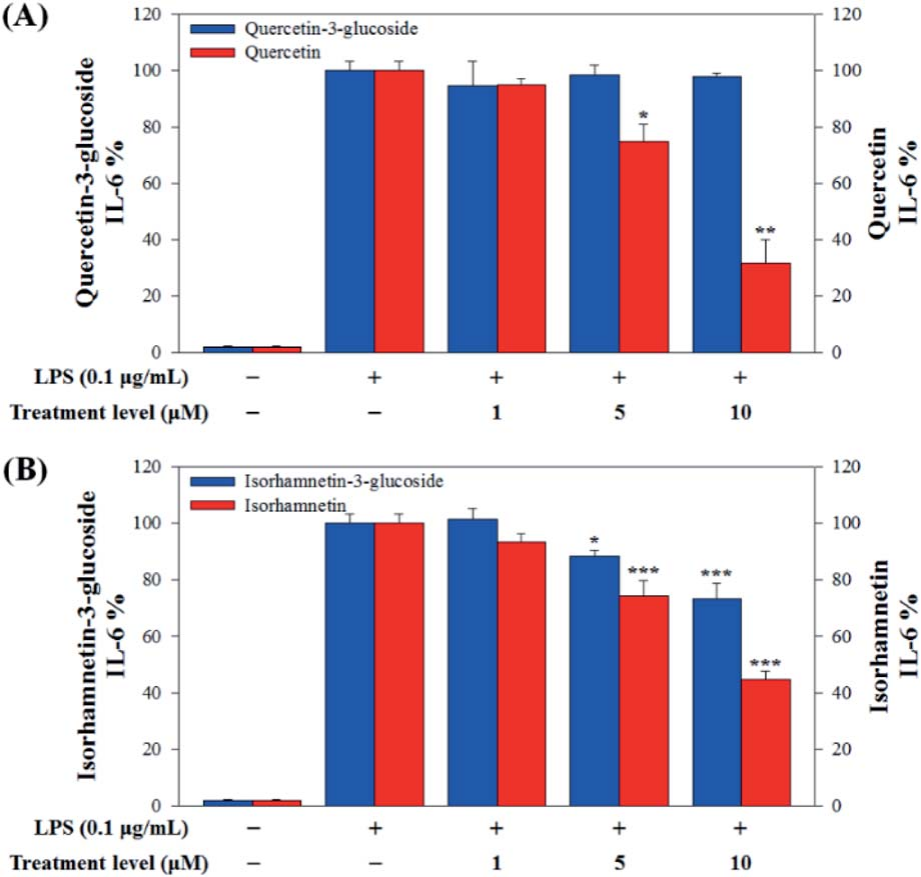
(A) Comparison of IL-6 production levels with quercetin-3-glucoside and quercetin and (B) isorhamnetin-3-glucoside and isorhamnetin. Values are the mean ± SD of three independent experiments. (*) *p* < 0.05, (**) *p* < 0.01, and (***) *p* < 0.001 indicate significant differences compared to the LPS-treated group.

In this study, to compare the anti-inflammatory effects of *S. herbacea* glycosides (Q3G and IR3G) and aglycones (quercetin and isorhamnetin), we evaluated the LPS-induced TNF-α (Fig. 6) and IL-6 levels (Fig. 7) in Raw 264.7 (KCLB 40071) mouse macrophage cells after Q3G, IR3G, quercetin and isorhamnetin treatments into macrophage cell culture media.

**Fig. 8:**
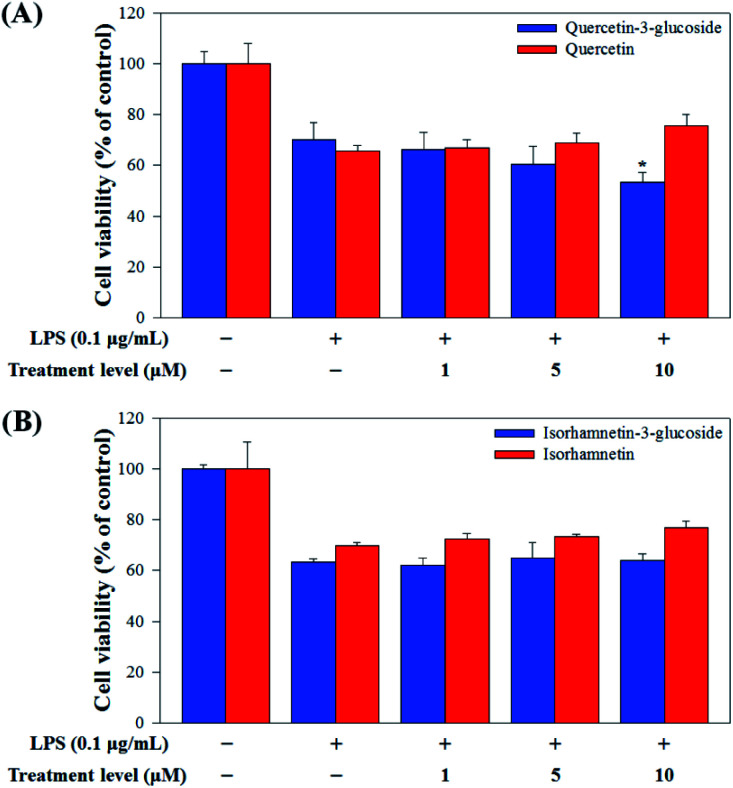
(A) Cell viability comparison of quercetin-3-glucoside and quercetin and (B) isorhamnetin-3-glucoside and isorhamnetin. Values are the mean ± SD of three independent experiments. (*) *p* < 0.05 indicates significant differences compared to the LPS-treated group.

Raw 264.7 cells were incubated with various concentrations of Q3G, quercetin, IR3G, and isorhamnetin (0, 1, 5, 10 μM) for 2 h. Then, except for the negative control group (no LPS), they were treated with LPS (0.1 μg mL^−1^) and incubated for 24 h. Finally, TNF-α and IL-6 production levels from Raw 264.7 cells were determined using an ELISA assay and a 3-(4,5-dimethylthiazol-2-yl)-2,5-diphenyltetrazolium bromide (MTT) assay was conducted to evaluate cell viability at the same time (Fig. 8).

Our results showed that Q3G increased TNF-α production, while quercetin decreased TNF-α production in a dose dependent manner. IR3G decreased TNF-α production but isorhamnetin decreased TNF-α production 1.5–4.6 times that of IR3G with the 1–10 μM treatment. Regarding IL-6 production, quercetin-3-glucose did not decrease IL-6 production with LPS. In contrast, quercetin did decrease TNF-α production in a dose dependent manner. Isorhamnetin exhibited greater inhibition of IL-6 production compared to IR3G; isorhamnetin was about 2.1 times higher at 1–10 μM. Cell viability was measured by MTT assay, with and without LPS. Q3G showed a trend of slightly decreasing cell viability, while IR3G, quercetin, and isorhamnetin did not affect cell viability at 1–10 μM. Thus, these results suggest that quercetin and isorhamnetin, produced by biotransformation using crude enzyme extract of AD011, inhibit inflammatory marker production more effectively than their precursors, Q3G and IR3G, respectively, in RAW 264.7 cells. The anti-inflammatory effect of aglycone molecules agrees with previous data from other groups.^41,66,68–73^

## Conclusions

In this study, we examined the conditions under which the maximum amount of flavonoid glycosides (Q3G and IR3G) from *S. herbacea* could be extracted by varying the solvent (methanol, 70% methanol and DI water) and drying conditions (heat and freeze drying) and screened probiotic strains to find one who's raw enzyme extract would efficiently convert these two flavonol glycosides into their aglycones (quercetin and isorhamnetin). Crude enzyme extracts from AD011 showed effective flavonol glycosides conversion properties. More than 85% of Q3G and IR3G were transformed to their aglycone forms within 2 h by AD011 crude enzyme extract and exhibited no structural degradation after 36 h of enzyme exposure. Transformed quercetin and isorhamnetin showed higher anti-inflammatory effects against RAW 264.7 macrophage induced by LPS than their mother molecules (Q3G and IR3G). Biotransformation of Q3G and IR3G in *S. herbacea* into their aglycone forms *via* the probiotic strain AD011 safely increased anti-inflammatory effects and bioavailability of *S. herbacea*. This work is the first to directly compare the anti-inflammatory effects of Q3G, IR3G, quercetin and isorhamnetin, and our work may contribute to the rapid and effective development of *S. herbacea* extracts or *S. herbacea*-containing functional foods that produce anti-inflammatory effects.

## Conflicts of interest

Hyung Jin Ahn, Hyun Ju You, Zhipeng Li, Deokyeong Choe, Tony Vaughn Johnston, and Seockmo Ku declare no conflict of interests. Myeong Soo Park is directly employed at BIFIDO Ltd. as CTO. Geun Eog Ji and Myeong Soo Park hold BIFIDO Ltd. stocks.

## Supplementary Material
